# The correlation between plasma cortisol levels and scores of psychological scales among patients with recurrent depressive disorder in Vietnam

**DOI:** 10.1371/journal.pone.0320776

**Published:** 2025-04-01

**Authors:** Tuan Van Nguyen, Eric Hahn, Thi Minh Tam Ta, Thi Phuong Mai Nguyen, Hoa Thi Nguyen, Quynh Thi Pham, Duc Minh Nguyen, Thi Thuy Chuyen Dieu, Thi Phuong Thao Nguyen, Ngan Thi Vuong, Tung Son Vu

**Affiliations:** 1 Department of Psychiatry, Hanoi Medical University, Hanoi, Vietnam; 2 National Institute of Mental Health, Bach Mai Hospital, Hanoi, Vietnam; 3 Department of Psychiatry and Psychotherapy, Charité - Universitätsmedizin, Campus Benjamin Franklin, Berlin, Germany; 4 Department of Psychiatry and Clinical Psychology, VNU University of Medicine and Pharmacy, Hanoi, Vietnam; 5 Department of Interventional Cardiology – Surgery, National Geriatric Hospital, Hanoi, Vietnam; 6 Laboratory of Biochemistry and Immunology, Vietnam National Cancer Hospital, Hanoi, Vietnam; Mae Fah Luang University School of Anti Aging and Regenerative Medicine, THAILAND

## Abstract

**Objectives:**

We aimed to examine levels of plasma cortisol in the morning and evening before and after standardised treatment, and analyze the correlation between these levels and scores of psychological assessment scales among patients with recurrent depressive disorder (PRDD) in Vietnam.

**Methods:**

From January 2020 to December 2021, a cross-sectional study was carried out at the National Institute of Mental Health in Bach Mai Hospital. After using a convenience sampling method, 109 consecutive patients met our criteria were recruited. We measured and analysed plasma cortisol levels in all participants at baseline (T0), two weeks after treatment (T1) and four weeks after treatment (T2). Spearman correlation was applied to assess the correlations between plasma cortisol at six different time and scores of six psychological assessment scales including HAM-D, HAM-A, BDI, SAS, DASS, and MMSE.

**Results:**

Among 109 PRDD, the percentage of subjects had high cortisol levels decreased during hospital treatment. At admission (T0), these figures were 4.76% in the morning and 8% in the evening. After 4-week treatment (T2), these figures declined to 1.32% and 3.09%, respectively. At T0, the morning cortisol concentrations had a positive correlation with the mean scores of HAM-A (r = 0.257), BDI (r = 0.251), and SAS (r = 0.276) (p ≤ 0.05), whereas the evening cortisol concentrations in the evening had a positive correlation with the mean scores of BDI (r = 0.197), SAS (r = 0.206), and Depression subscale of DASS (r = 0.252) (p ≤ 0.05). At T2, we did not detect any correlation between morning or evening cortisol levels and six test scores.

**Conclusion:**

The utilization of psychological measures for monitoring purposes can facilitate the assessment of alterations in cortisol levels among individuals experiencing recurrent depression, hence aiding in the treatment of depression in this population. Additional investigation using a more extensive sample size is required to furnish additional substantiation on this matter in Vietnam.

## Introduction

Depression is a chronic mental disorder and one of the primary contributors to global impairment. Depression is distinct from typical fluctuations in mood and emotions related to daily existence. It has the potential to impact several facets of life, encompassing familial, social, and communal ties [1]. According to a recent report by the World Health Organization (2017), the prevalence of depression among the global population in 2015 was 4.4% [2]. The overall estimated population of individuals afflicted with depression experienced a growth of 18.4% from 2005 to 2015, representing the overall growth of the worldwide population with depression. Notably, approximately half of this increase was concentrated in Southeast Asia and the Western Pacific region [2]. Depressive disorder stands out for its chronic and recurrent nature [3,4]. Approximately 50% of individuals who have successfully overcome depression still experience one or more future bouts of depression, ranging from 3 to 5 [5–7]. In a prospective study conducted by Mueller et al. (1999), it was found that out of the 380 depressed patients who had totally recovered, 85% of them experienced relapses of at least one depressive episode after a period of 15 years [8]. This has substantial implications for both personal and national health. Approximately 15 to 25% of individuals with recurrent depression are predicted to experience a fall-winter pattern of episodes [9], indicating that this type of depression is highly prevalent.

Depression is linked to neuroendocrine disorders. Disruptions in cortisol release are among the key causes of concern [10]. Extended exposure to stress, a challenging risk factor to evade to depression patients, induces physiological alterations in the body, including the activation of the hypothalamic-pituitary-adrenal axis (HPA) [10], a neuroendocrine system that primarily regulates the body’s stress response [11]. Dysregulation of the HPA axis, often reflected in abnormal cortisol responses, has been implicated in various psychiatric disorders [12]. A considerable amount of research indicates that its activity is greatly increased in patients suffering from depression, compared to healthy controls [13,14], so depression is associated with hyperfunction of the HPA axis. The activation of HPA leads to an increased release of cortisol, a crucial steroid hormone produced by the adrenal cortex [10]. The Dexamethasone Suppression Test (DST) has been studied as a potential biomarker in psychiatry, specifically for its role in assessing HPA axis dysregulation, through measuring the body’s ability to regulate cortisol production following administration of dexamethasone, a synthetic glucocorticoid [15]. Cortisol exerts a substantial impact on various physiological processes, including metabolism, gene expression, and the central nervous system, hence exerting a profound influence on the mental well-being of individuals [10]. It is reported that approximately 50% of individuals who have recently been diagnosed with depression exhibit an elevated level of cortisol secretion [16]. As a results, cortisol is widely regarded as a prominent biomarker for indicators of anxiety disorders and depression [10].

Vietnam, a nation characterised by limited human resources for mental health, exhibited a prevalence rate of 14.2% for 10 prevalent mental diseases, with depression accounting for 2.45% of these cases [17]. Although UNICEF, MOLISA, and ODI have made progress and provided support, care and response services in Vietnam have not yet adequately addressed the demand for primary mental health care and depression treatment [18]. Currently, there was a lack of reporting on aspects revolving around people with recurrent depression and their cortisol levels in Vietnam. Furthermore, the linear relationship between plasma cortisol level and psychological assessment scales were not taken account in patients with recurrent depressive disorder (PRDD) in previous studies in Vietnam. The aim of this study was therefore to evaluate morning and evening plasma cortisol concentrations before and after standardised treatment, and investigate the correlation between these levels and psychological assessment scales and measures among patients with recurrent depressive disorder in hospital based mental health care setting in Vietnam.

## Methods

### Study design, location and time

A hospital-based cross-sectional study was carried out at National Institute of Mental Health (NIMH), Bach Mai Hospital, Hanoi, Vietnam. The research time ranged from January 2020 to December 2021. The period of collecting data was between May 2020 and December 2021. NIMH is among the most historical governmental hospitals in Vietnam. The psychiatric clinic at NIMH offers services for mentally ill patients in both the outpatient and inpatient departments, catering to the population mostly in North Vietnam.

### Study population

Participants who (a) fulfilled ICD-10 criteria [19] for the diagnosis of recurrent depressive disorder, (b) were receiving inpatient treatment with follow up plan at our institution, and (c) voluntarily agreed to participate in the study were included in the study. We excluded patients suffering from any of the following conditions: (a) severe medical conditions such as traumatic brain injury, malignancy or emergency disease conditions; or (b) endocrine diseases causing hyper-and hypoactivity of the adrenal cortex; or (c) medical condition that interfered with communication that was not caused by depression.

### Sample size and sampling

We applied the sample size formula to estimate a proportion in a population: *n =* $Z_{\left(1-\frac{\alpha }{2}\right)}^{2}$ $\frac{p\left(1-p\right)}{{\left(p.\varepsilon \right)}^{2}}$

In which, n is the required sample size, *α* is level of statistical significance (choose *α* =  0.05 corresponding to 95%CI), p =  0.46 (which was recorded as the increase rate in cortisol concentration according to Jadwiga Piwowarska et al., 2009 [20]), ε =  0.3 (desired error between study sample and population). Finally, the calculated sample size was 51. Then, we added 20% of the participants in case they may be the possibility of refusals and/or losses. Finally, a total of 109 patients with a diagnosis of recurrent depressive disorder were recruited in the study. A convenience sampling method was applied until the minimum sample size was reached.

However, at 6 time points, the sample size changed because we eliminated missing values. Therefore, the sample size at 8 a.m. T0, 8 a.m. T1, 8 a.m. T2, 8 p.m. T0, 8 p.m. T1, and 8 p.m. T2 were 63, 71, 76, 100, 105, and 97, respectively.

### Study variables

(1) **Dependent variables**

The main study outcome variables or dependent variables were presented as quantitative values, known to be cortisol concentrations (nmol/L) at the time of admission immediately after the diagnosis (T0), two weeks after treatment (T1) and four weeks after treatment (T2), corresponding to two times of 8 a.m. (morning) and 8 p.m. (evening) in one day.

(2) **Inpendent variables**

- Socio-demographic characteristics include gender, living area, religion, ethnicity, education levels, occupation.
- The scores of psychological tests including Hamilton Depression Rating Scale (HAM-D), Hamilton Anxiety Rating Scale (HAM-A), Beck Depression Inventory (BDI), Zung Self-Rating Depression Scale (SDS), Depression Anxiety Stress Scale (DASS), Mini-Mental State Examination (MMSE).

- HAM-D is a 17-domain scale to assess the level of depression, with the total score ranging from 0 to 54. Greater scores indicate more severe depression.
- HAM-A is used as a standard scale for anxiety measurement, with the total score of 14 categories being between 0 and 56. Higher scores reflect increased anxiety severity.
- BDI is the gold standard among self-rating scales for depression. The version commonly used in clinical practice consists of 21 questions, with scores ranging from 0 to 63 points. Higher scores correspond to more intense depression.
- SDS is a self-report questionnaire to assess the severity of anxiety disorders. The scale consists of 20 components, and the total score varies between 20 and 80 points. Higher scores indicate greater degree of anxiety.
- DASS includes 42 questions categorised into three subscales: depression, anxiety, and stress. Each subscale consists of 14 questions, with a maximum score of 42 points for each subscale. Scores on each subscale of depression, anxiety, and stress rise in accordance with the severity level.
- MMSE is commonly utilised to evaluate cognitive capabilities. The total score ranges from 0 to 30 points, with lower scores correspond to higher levels of cognitive impairment.

### Sample collection procedure

The timing of a cortisol level test is important because cortisol levels change throughout the day. Following being enrolled in the study, the entire cohort of study patient was taken the venous blood sample for the measurement of plasma cortisol concentrations at two different time points, at the time of admission immediately after the diagnosis (T0), two weeks after treatment (T1) and four weeks after treatment (T2). At each time point, blood sample of them was taken twice a day of 8 a.m. in the morning and 8 p.m. in the evening. The sample was collected by diploma nurses and standard operating procedure of nursing practice was strictly followed during sample collection of Bach Mai Hospital.

Patients are allowed to rest in bed for 5–10 minutes before blood collection. Each specimen includes 5 ml of whole blood. The nurse takes blood into a test tube (containing Heparin, which has an anticoagulant effect, separates plasma for testing) and shakes the blood well in the test tube 8–10 times. Then, the sample was sent to laboratory (Department of Biochemistry, Bach Mai Hospital) for analysis. After verified by biochemistry technicians, the sample was stored at 2–8°C. Then, the collected sample were immediately centrifuged, before being placed into the specimen racks and and put it into the analyzer. Finally, COBAS 8000 operating procedure was complied until cortisol test results appeared.

The reference ranges for normal limits of Cortisol concentration measured with the Cobas 8000 are 171–536 (nmol/L) and 64–327 (nmol/L) for 8 a.m. in the morning and 8 p.m. in the evening, respectively.

### Data analysis

The data obtained were entered in EpiData 3.1 (The EpiData Association, Odense, Denmark), and then were coded appropriately before being imported to Stata® 16.0 (StataCorp LLC, College Station, TX, USA) for analysis. Descriptive statistical analysis was used to describe characteristics among study patients. We expressed frequency and percentage for qualitative variable, and mean and standard deviation (SD) for quantitative variables. Regarding to inference statistics, we analyzed the correlations between serum cortisol concentrations in the morning and evening and the mean scores of six psychological tests at three time points (T0, T1, and T2) using Spearman. The r-valued correlation coefficient varied between −1 and + 1. The levels of correlation are as follows: −1 < r <  0 denotes negative correlation, 0 <  r < + 1 denotes positive correlation, and r =  ± 1 denotes perfect correlation. Furthermore, the correlation level is expressed as follows: ± 0.0 to ± 0.3 shows negligible correlation, ± 0.3 to ± 0.5 shows low correlation, ± 0.5 to ± 0.7 shows moderate correlation, ± 0.7 to ± 0.9 shows high correlation, ± 0.9 to ± 1.0 shows very high correlation [19]. A p-value < 0.05 was considered statistically significant.

### Ethical statement

Before participating in the study, study subjects were provided written consent form. Participation was voluntary, and anonymity was assured. All information collected was confidential and is only used for research purposes. Our research proposal and protocol were conducted by the Declaration of Helsinki, and approved by the Hanoi Medical University Institutional Ethical Review Board (HMU IRB) (IRB-VN01.001/IRB00003121/FWA 00004148) with the Approval No: 65/GCN-HĐĐĐNCYSH-ĐHYHN; Day: 16 April 2020.

## Results

Table 1 presents the characteristics of the study population. The mean age is 48.66 ±  15.07 (years). Most participants were female (72.48%), lived with family (97.25%), in the rural area (65.14%) and had no religion (91.74%). The majority of participants were married (79.82%). Economically, 86.24% of the participants were at the middle class. 44.95% of the participants possessed a level of below high school such as primary or secondary school, while 30.28% had above high school education, and 22.94% were at high school level. Regarding occupation, nearly 40% of subjects are farmers, accounting for the largest proportion.

**Table 1:** Socio-demographic characteristics of study subjects (n = 109).

Characteristics		n (%)	
Age (mean ± SD)		48.66 ± 15.07	
Gender	Female	79	72.48
	Male	30	27.52
Living area	Rural	71	65.14
	Urban	38	34.86
Living circumstances	Living with family (parents, spouse, children)	106	97.25
	Living alone	3	2.75
Religion	No	100	91.74
	Yes	9	8.26
Marital status	Single	11	10.09
	Married	87	79.82
	Divorced	4	3.67
	Widow	7	6.42
Financial situation	Poor	13	11.93
	Medium	94	86.24
	Wealthy	2	1.83
Education levels	Illiteracy	2	1.83
	Below high school	49	44.95
	High school	25	22.94
	Above high school	33	30.28
Occupation	Farmer	42	38.53
	Worker	9	8.26
	Staff	12	11.01
	Student	3	2.75
	Businessmen/women	9	8.26
	Retire	13	11.93
	Unknown	21	19.26

**Fig 1:**
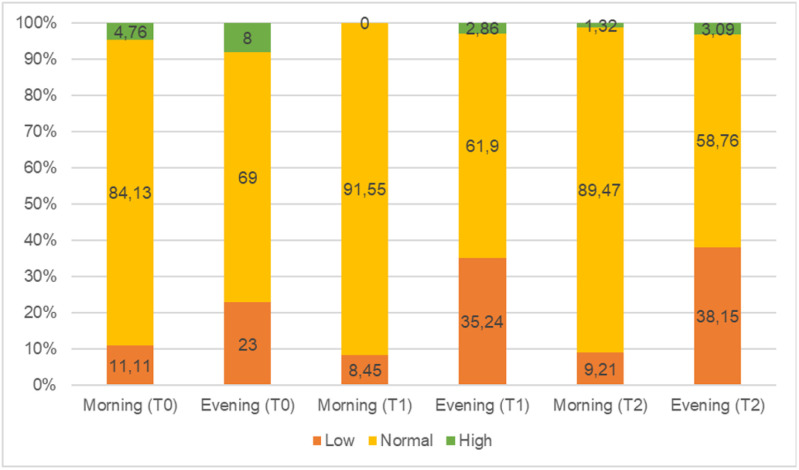
The proportion of three groups of cortisol concentrations in the morning and evening at eeach time point (T0, T1, T2) among patients with recurrent depressive disorder. Note: The numbers of samples calculated at each stage after removing missing values are 63 (T0 – in the morning), 100 (T0 – in the evening), 71 (T1 – in the morning), 105 (T1 – in the evening), 76 (T2 – in the morning), and 97 (T2 – in the evening).

Figure 1 depicts the percentage of three groups of cortisol concentrations at each time among study subjects. At each time point, the proportion of participants whose cortisol levels were within the normal range was the highest. Over the period of 4-week treatment from admission, the percentage of people having high morning cortisol levels declined from 4.76% at T0 to 1.32% at T2, whereas those having high evening cortisol levels witnessed a decrease from 8% at T0 to 3.09% at T2.

Table 2 shows the Spearman correlation between cortisol concentrations in the morning or evening and the average scores of six scales including HAM-D, HAM-A, BDI, SAS, DASS and MMSE at admission (T0). At T0, the cortisol concentrations in the morning had a positive correlation with the mean scores of HAM-A (r = 0.257), BDI (r = 0.251), and SAS (r = 0.276) (p ≤ 0.05). Meanwhile, the cortisol concentrations in the evening had a positive correlation with the mean scores of BDI (r = 0.197), SAS (r = 0.206), and Depression subscale of DASS (r = 0.252) (p ≤ 0.05).

**Table 2:** Spearman correlation between cortisol concentrations in the morning or evening and the average scores of six scales at admission (T0).

Scales	Mean score (mean ± SD)	Morning cortisol concentrations (n = 63)		Evening cortisol concentrations (n = 100)	
		r	p	r	p
HAM-D	21.35 ± 8.60	0.231	0.068	0.162	0.107
HAM-A	21.90 ± 11.32	0.257	0.042^*^	0.063	0.532
BDI	28.32 ± 14.31	0.251	0.048^*^	0.197	0.05^*^
SAS	43.46 ± 10.24	0.276	0.028^*^	0.206	0.04^*^
DASS – Depression subscale	19.66 ± 12.75	0.174	0.173	0.252	0.011^*^
DASS – Anxiety subscale	17.76 ± 10.84	0.144	0.262	0.121	0.229
DASS - Stress subscale	20.53 ± 10.93	0.098	0.445	0.166	0.098
MMSE	26.12 ± 3.53	0.054	0.674	0.019	0.854

* p ≤ 0.05: statistically significant

Table 3 represents the Spearman correlation between cortisol concentrations in the morning or evening and the average scores of six scales including HAM-D, HAM-A, BDI, SAS, DASS and MMSE at two weeks after treatment (T1). At T1, the cortisol concentrations in the morning had a positive correlation with the mean scores of HAM-A (r = 0.292), BDI (r = 0.396), and DASS (r =  0.32 for depression subscale, r = 0.309 for anxiety subscale, r = 0.291 for stress subscale) (p ≤ 0.05). Meanwhile, the cortisol concentrations in the evening had no correlation with any score of tests.

**Table 3:** Spearman correlation between serum cortisol concentrations in the morning or evening and the average scores of six scales at two weeks after treatment (T1).

Scales	Mean score (mean ± SD)	Morning cortisol concentrations (n = 71)		Evening cortisol concentrations (n = 105)	
		r	p	r	p
HAM-D	17.72 ± 9.44	0.213	0.075	−0.059	0.549
HAM-A	17.47 ± 12.19	0.292	0.014^*^	−0.060	0.541
BDI	20.77 ± 14.55	0.396	0.001^*^	0.035	0.723
SDS	39.74 ± 10.26	0.207	0.086	−0.115	0.246
DASS – Depression subscale	14.68 ± 11.86	0.320	0.007^*^	0.059	0.552
DASS – Anxiety subscale	13.50 ± 10.16	0.309	0.009^*^	−0.025	0.801
DASS - Stress subscale	15.24 ± 10.62	0.291	0.014^*^	−0.057	0.561
MMSE	26.74 ± 3.03	-0.163	0.175	−0.056	0.573

* p ≤ 0.05: statistically significant

Table 4 shows the Spearman correlation between cortisol concentrations in the morning and evening and the average scores of six scales including HAM-D, HAM-A, BDI, SAS, DASS and MMSE four weeks after treatment (T2). We did not detect any correlation between morning or evening cortisol levels and test scores at T2.

**Table 4:** Spearman correlation between serum cortisol concentrations in the morning or evening and the average scores of six scales at four weeks after treatment (T2).

Scales	Mean score (mean ± SD)	Morning cortisol concentrations (n = 76)		Evening cortisol concentrations (n = 97)	
		r	p	r	p
HAM-D	13.13 ± 8.60	0.066	0.569	−0.147	0.151
HAM-A	12.91 ± 10.98	−0.089	0.441	−0.154	0.131
BDI	14.17 ± 11.61	0.089	0.444	−0.085	0.407
SAS	34.99 ± 8.45	−0.215	0.063	−0.177	0.083
DASS – Depression subscale	10.24 ± 9.58	−0.001	0.993	−0.146	0.154
DASS – Anxiety subscale	10.38 ± 9.10	−0.039	0.741	−0.154	0.131
DASS - Stress subscale	11.60 ± 9.70	−0.026	0.822	−0.117	0.253
MMSE	27.32 ± 3.27	0.099	0.396	0.123	0.229

* p ≤ 0.05: statistically significant

## Discussion

To the best of our knowledge, this study is one of the first in Vietnam recurrent depressive disorder to evaluate cortisol concentrations in the morning and evening among PRDD. Our study contributes to the body of evidence regarding cortisol concentrations and their correlations with psychiatric tests among patients in Vietnam who are suffering from recurrent depression. As a result, doctors, clinicians and researchers will have a solid foundation upon which to develop effective treatments for the patients.

The findings of our investigation indicate the increase percentage of study subjects having low plasma cortisol concentrations in the morning following hospital treatment. Our finding partly coincides with the outcomes reported by Nguyen Huu Thien (2019), who conducted a study including 61 individuals diagnosed with severe depressive disorder, when his investigation revealed a statistically significant decline in cortisol concentrations both prior to and during treatment [20]. Our study suggests that clinical intervention may contribute to the decrease in cortisol levels among individuals diagnosed with depression, though other factors such as natural hormonal regulation over time could also be involved. The phenomenon of cortisol hypersecretion has been proposed as a potential biological risk factor for depression, as indicated by Goodyer et al. (2000) [21], because depressed patients exhibited elevated cortisol levels [22]. An earlier research showed that a higher cortisol level is associated with more severe depressed symptoms [23]. However, differences in study methodologies, populations, and healthcare systems should be considered when interpreting these findings. Variations in treatment protocols, access to healthcare, and cultural perceptions of mental health between Vietnam and other settings may influence cortisol responses to treatment. Therefore, the objective of therapy for individuals with depression is to regulate cortisol levels, rather than solely aiming for a reduction, because reduction in cortisol levels could reflect a return to baseline rather than a direct effect of treatment.

Cortisol secretion abnormalities can lead to mental diseases and are among the various hormonal disorders that often coexist with these symptoms, such as depression [10]. In terms of HAM-D, one of the tests reprenting levels of depression, our study indicated that there was no significant correlation observed between morning and evening cortisol levels throughout admission and after-treatment durations, and the HAM-D score. These findings align with the recent research conducted by Alenko et al. (2020), which also observed no statistically significant associations between baseline and endline blood cortisol levels and HAM-D scores [24]. Regarding to other depression test, our findings indicted that cortisol levels at admission and 2-week after treatment had a positive significantly correlation with BDI score. This was similar to Tse and Bond (2004) when they proved that there was a strong correlation observed between cortisol concentrations and BDI scores [22].

There exists a correlation between stress and heightened levels of anxiety, diminished levels of life satisfaction, and elevated rates of mental disease [25]. Lenze et al. (2011) provided evidence indicating that the treatment of anxiety disorders in older persons leads to a decrease in high cortisol levels [26]. In our study, we found that positive significant correlations between morning cortisol levels at T0 and T1 and HAM-A score, whose scale indicated levels of anxiety. In a cross-sectional study, Lawson et al. (2009) demonstrated the same, when showed a positive correlation between general cortisol levels and HAM-A score [27]. In relation to SAS, we found that only morning and evening cortisol levels at the admission had a correlation with SAS score, though this correlation was negligible. Meanwhile, a previous investigation carried out on women experiencing infertility revealed a significant association between the morning cortisol level and the SAS score among this population [28]. Further investigation is required to elucidate the correlation between cortisol levels and SAS scores.

Enhanced comprehension of the intricate connections among cortisol, cognition, and dementia holds the potential to unveil novel avenues for prevention and treatment strategies centred on the HPA axis. Indeed, multiple prior research have demonstrated that disrupted functioning of the HPA axis, specifically elevated cortisol levels in older individuals, is linked to a heightened susceptibility to dementia and Alzheimer’s disease (AD) [29–32]. An increase in cortisol levels was found to be correlated with reduced cognitive performance across various domains, including episodic memory, executive functioning, language, spatial memory, processing speed, and social cognition [33]. In this study, our findings did not find any correlation between cortisol levels at any time and MMSE score, whose scale represents levels of cognitive impairment. In contrast, a previous study carried out on elderly individuals who underwent hip fracture surgery revealed a negative correlation between plasma cortisol levels and MMSE score [34]. The reason for this difference may be due to our small sample size.

Several limitations were observed in this research. First, it should be noted that the sample size employed in our study was insufficient to establish a significant association between plasma cortisol levels and potential alterations in psychometric scores. Second, as this study was based on a cross-sectional design, it did not track cortisol changes within the same individuals over time, difficult to determine whether observed differences in cortisol levels were due to treatment effects, natural remission, or other external factors. To minimize variability, we ensured that all cortisol measurements were taken at fixed time points (8 AM and 8 PM) and analyzed using a standardized laboratory method. Finally, because of COVID-19 pandemic, the exclusion of a control group was attributed to the research team’s inadequate resources. The absence of a control group poses challenges in assessing the temporal decline in plasma cortisol levels. To mitigate this limitation, we controlled for potential confounders, including age, sex, and comorbidities, in our statistical analyses. However, we acknowledge that factors such as natural remission and the hospital environment may still influence cortisol levels. Therefore, we highly recommend that future studies conduct similar studies on a larger scale with larger sample sizes or longitudinal designs with control groups to strengthen causal interpretations and fill our above research gaps.

## Conclusion

The positive indication of effective treatment is shown when there was a decrease in the proportion of patients with recurrent depression having high cortisol levels after four-week treatment. The monitoring of psychological scale scores can be important in assessing alterations in cortisol levels among individuals experiencing recurrent depression, hence facilitating the treatment of depression in this population. However, given the transient and weak correlations observed, cortisol alone may not serve as a definitive biomarker for depression, so stronger evidence is needed to establish its clinical utility.

## Supporting information

S1 File Data. (XLSX)

## References

[1] World Health Organization. Depressive disorder (depression) [Internet]. [cited on 30 Mar 2024]. Available from: https://www.who.int/news-room/fact-sheets/detail/depression

[2] World Health Organization. Depression and other common mental disorders: global health estimates [Internet]. Report No.: WHO/MSD/MER/2017.2. World Health Organization; 2017. Available from: https://apps.who.int/iris/handle/10665/254610

[3] Vos T, Haby MM, Barendregt JJ, Kruijshaar M, Corry J, Andrews G. The burden of major depression avoidable by longer-term treatment strategies. Arch Gen Psychiatry. 2004;61(11):1097–103. doi: 10.1001/archpsyc.61.11.1097 15520357

[4] Murray CJ, Lopez AD. Global mortality, disability, and the contribution of risk factors: global burden of disease study. Lancet. 1997;349(9063):1436–42. doi: 10.1016/S0140-6736(96)07495-8 9164317

[5] Birmaher B, Arbelaez C, Brent D. Course and outcome of child and adolescent major depressive disorder. Child Adolesc Psychiatr Clin N Am. 2002;11(3):619–37, x. doi: 10.1016/s1056-4993(02)00011-1 12222086

[6] van Weel-Baumgarten E, van den Bosch W, van den Hoogen H, Zitman FG. Ten year follow-up of depression after diagnosis in general practice. Br J Gen Pract. 1998;48(435):1643–6. 10071395 PMC1313237

[7] Wilson I, Duszynski K, Mant A. A 5-year follow-up of general practice patients experiencing depression. Fam Pract. 2003;20(6):685–9. doi: 10.1093/fampra/cmg611 14701893

[8] Mueller TI, Leon AC, Keller MB, Solomon DA, Endicott J, Coryell W, et al. Recurrence after recovery from major depressive disorder during 15 years of observational follow-up. Am J Psychiatry. 1999;156(7):1000–6. doi: 10.1176/ajp.156.7.1000 10401442

[9] Thase ME. Comparison between seasonal affective disorder and other forms of recurrent depression. Seasonal affective disorders and phototherapy. New York, NY, US: Guilford Press; 1989. p. 64–78.

[10] Dziurkowska E, Wesolowski M. Cortisol as a biomarker of mental disorder severity. J Clin Med. 2021;10(21):5204. doi: 10.3390/jcm10215204 34768724 PMC8584322

[11] Berardelli I, Serafini G, Cortese N, Fiaschè F, O’Connor RC, Pompili M. The involvement of hypothalamus-pituitary-adrenal (HPA) axis in suicide risk. Brain Sci. 2020;10(9):653. doi: 10.3390/brainsci10090653 32967089 PMC7565104

[12] Holsboer F. The corticosteroid receptor hypothesis of depression. Neuropsychopharmacology. 2000;23(5):477–501. doi: 10.1016/S0893-133X(00)00159-7 11027914

[13] Stetler C, Miller GE. Depression and hypothalamic-pituitary-adrenal activation: a quantitative summary of four decades of research. Psychosom Med. 2011;73(2):114–26. doi: 10.1097/PSY.0b013e31820ad12b 21257974

[14] Zunszain PA, Anacker C, Cattaneo A, Carvalho LA, Pariante CM. Glucocorticoids, cytokines and brain abnormalities in depression. Prog Neuropsychopharmacol Biol Psychiatry. 2011;35(3):722–9. doi: 10.1016/j.pnpbp.2010.04.011 20406665 PMC3513408

[15] Schumacher MM, Santambrogio J. Cortisol and the dexamethasone suppression test as a biomarker for melancholic depression: a narrative review. J Pers Med. 2023;13(5):837. doi: 10.3390/jpm13050837 37241007 PMC10223878

[16] Butterworth JF, Wasnick JD, Mackey DC. Morgan and Mikhail’s clinical anesthesiology. 6th ed. McGraw-Hill Education; 2018.

[17] World Health Organization. Mental health in Viet Nam [Internet]. [cited on 30 Mar 2024]. Available from: https://www.who.int/vietnam/health-topics/mental-health

[18] Vuong DA, Van Ginneken E, Morris J, Ha ST, Busse R. Mental health in Vietnam: burden of disease and availability of services. Asian J Psychiatr. 2011;4(1):65–70. doi: 10.1016/j.ajp.2011.01.005 23050918

[19] Hinkle DE, Wiersma W, Jurs SG. Applied statistics for the behavioral sciences. Houghton Mifflin; 2003.

[20] Nguyen HT. Plasma cortisol concentration in patients with major depressive disorder; 2019.

[21] Goodyer IM, Herbert J, Tamplin A, Altham PM. Recent life events, cortisol, dehydroepiandrosterone and the onset of major depression in high-risk adolescents. Br J Psychiatry. 2000;177:499–504. doi: 10.1192/bjp.177.6.499 11102323

[22] Tse WS, Bond AJ. Relationship between baseline cortisol, social functioning and depression: a mediation analysis. Psychiatry Res. 2004;126(3):197–201. doi: 10.1016/j.psychres.2004.02.002 15157746

[23] Zobel AW, Nickel T, Sonntag A, Uhr M, Holsboer F, Ising M. Cortisol response in the combined dexamethasone/CRH test as predictor of relapse in patients with remitted depression. a prospective study. J Psychiatr Res. 2001;35(2):83–94. doi: 10.1016/s0022-3956(01)00013-9 11377437

[24] Alenko A, Markos Y, Fikru C, Tadesse E, Gedefaw L. Association of serum cortisol level with severity of depression and improvement in newly diagnosed patients with major depressive disorder in Jimma medical center, Southwest Ethiopia. PLoS One. 2020;15(10):e0240668. doi: 10.1371/journal.pone.0240668 33064754 PMC7567351

[25] Roos LG, Levens SM, Bennett JM. Stressful life events, relationship stressors, and cortisol reactivity: the moderating role of suppression. Psychoneuroendocrinology. 2018;89:69–77. doi: 10.1016/j.psyneuen.2017.12.026 29331801 PMC5878721

[26] Lenze EJ, Mantella RC, Shi P, Goate AM, Nowotny P, Butters MA, et al. Elevated cortisol in older adults with generalized anxiety disorder is reduced by treatment: a placebo-controlled evaluation of escitalopram. Am J Geriatr Psychiatry. 2011;19(5):482–90. doi: 10.1097/JGP.0b013e3181ec806c 20808146 PMC3424606

[27] Lawson EA, Donoho D, Miller KK, Misra M, Meenaghan E, Lydecker J, et al. Hypercortisolemia is associated with severity of bone loss and depression in hypothalamic amenorrhea and anorexia nervosa. J Clin Endocrinol Metab. 2009;94(12):4710–6. doi: 10.1210/jc.2009-1046 19837921 PMC2795653

[28] Chai Y, Li Q, Wang Y, Niu B, Chen H, Fan T, et al. Cortisol dysregulation in anxiety infertile women and the influence on IVF treatment outcome. Front Endocrinol (Lausanne). 2023;14:1107765. doi: 10.3389/fendo.2023.1107765 37383394 PMC10299854

[29] Lupien SJ, Nair NP, Brière S, Maheu F, Tu MT, Lemay M, et al. Increased cortisol levels and impaired cognition in human aging: implication for depression and dementia in later life. Rev Neurosci. 1999;10(2):117–39. doi: 10.1515/revneuro.1999.10.2.117 10658955

[30] Rothman SM, Mattson MP. Adverse stress, hippocampal networks, and Alzheimer’s disease. Neuromolecular Med. 2010;12(1):56–70. doi: 10.1007/s12017-009-8107-9 19943124 PMC2833224

[31] Ennis GE, An Y, Resnick SM, Ferrucci L, O’Brien RJ, Moffat SD. Long-term cortisol measures predict Alzheimer disease risk. Neurology. 2017;88(4):371–8. doi: 10.1212/WNL.0000000000003537 27986873 PMC5272965

[32] Notarianni E. Cortisol: Mediator of association between Alzheimer’s disease and diabetes mellitus?. Psychoneuroendocrinology. 2017;81:129–37. doi: 10.1016/j.psyneuen.2017.04.008 28458232

[33] Ouanes S, Popp J. High cortisol and the risk of dementia and Alzheimer’s disease: a review of the literature. Front Aging Neurosci. 2019;11:43. doi: 10.3389/fnagi.2019.00043 30881301 PMC6405479

[34] Ji M-H, Shen J-C, Gao R, Liu X-Y, Yuan H-M, Dong L, et al. Early postoperative cognitive dysfunction is associated with higher cortisol levels in aged patients following hip fracture surgery. J Anesth. 2013;27(6):942–4. doi: 10.1007/s00540-013-1633-5 23666452

